# Assessing potential collateral effects on amphibians from insecticide applications for flea control and plague mitigation

**DOI:** 10.1371/journal.pone.0320382

**Published:** 2025-05-12

**Authors:** David A. Eads, Susan A. Shriner, Jeremy W. Ellis, Paul M. Cryan, Michelle L. Hladik, Gregory P. Dooley, Erin Muths

**Affiliations:** 1 U.S. Geological Survey, Fort Collins Science Center, Fort Collins, Colorado, United States of America‌; 2 National Wildlife Research Center, U.S. Department of Agriculture, Animal and Plant Health Inspection Service, Wildlife Services, Fort Collins, Colorado, United States of America; 3 U.S. Geological Survey, California Water Science Center, Sacramento, California, United States of America; 4 Department of Environmental and Radiological Health Sciences, College of Veterinary Medicine and Biomedical Sciences, Colorado State University, Fort Collins, Colorado, United States of America; Southeastern Louisiana University, UNITED STATES OF AMERICA

## Abstract

Ideal disease mitigation measures for wildlife are safe and benign for target species, non-target organisms, the environment, and humans. Identifying collateral (i.e., unintended) effects is a key consideration in implementing such actions. Deltamethrin dust and fipronil-laced baits represent a group of insecticides that target fleas (pulicides) and are used to control flea (Siphonaptera) vectors of the plague bacterium *Yersinia pestis* to protect prairie dogs (*Cynomys* spp.) and their plague-susceptible obligate predators, endangered black-footed ferrets (*Mustela nigripes*). A variety of animals use prairie dog burrows as refuge, which potentially exposes them to deltamethrin, and to fipronil and its metabolites in fecal pellets excreted by prairie dogs and other mammals that have eaten fipronil baits. We assessed the potential effects of deltamethrin and fipronil residues on survival, body mass, and activity of western tiger salamanders (*Ambystoma mavortium*), a burrow-inhabiting amphibian. Pulicides were applied at realistic concentrations in mesocosms mimicking burrows. Treatments included (1) deltamethrin dust and non-treated prairie dog fecal pellets, (2) prairie dog fecal pellets containing fipronil and fipronil sulfone, and (3) un-treated prairie dog fecal pellets as controls. All 29 salamanders survived the experiment. We did not detect pulicide residues in any control salamanders. Fipronil sulfone was detected in tissues from 3 of 10 salamanders in the fipronil treatment and deltamethrin was detected in tissues from 9 of 11 salamanders in the deltamethrin treatment. Salamanders were observed outside of burrows more frequently after treatments than before. Deltamethrin concentrations in whole body samples correlated positively with the amount of time salamanders were inside burrows. Acute, lethal effects were not detected, but uptake of deltamethrin and, to a lesser extent fipronil sulfone, into salamander tissues indicated the potential for long-term effects on this non-target species. Identifying potential collateral effects is an important aspect of evaluating mitigation actions implemented to protect endangered species.

## Introduction

Conservation actions are often aimed at ameliorating or removing stressors affecting organisms of concern. Such actions may target a particular species of concern directly (e.g., via translocation, vaccination), indirectly by targeting other animals it depends on for survival (e.g., by increasing food sources), or both (e.g., by habitat improvement or decreasing disease vectors). The control of plague in prairie dogs (*Cynomys* spp.), an obligate food source for the endangered black-footed ferret (*Mustela nigripes*; hereafter ferret), targets reduction of fleas (Siphonaptera) that vector the plague bacterium *Yersinia pestis*. This management with pulicides (i.e., agents to control fleas) is often highly effective in achieving identified conservation results (e.g., persistence of critical food and habitat for the ferret, and protection of the ferrets themselves [1]), but may have unintended consequences to non-target species [2,3]. Collateral effects are sometimes less recognized and may be more likely in taxa that have commensal relationships with the organism targeted for conservation action (e.g., ambystomatid salamanders [4]).

Using pulicides in prairie dog colonies is a conservation action directed at protecting prairie dogs, ferrets, and associated wildlife [5,6]. Consistent implementation of this management action began in the early 2000s and is used successfully at black-footed ferret reintroduction sites across western North America [5–9]. In 2018 for example, biologists working with ferrets in the United States treated at least 6,274 ha (~ 24 sq. mi.) of prairie dog habitat with deltamethrin dust (DeltaDust^®^, 0.05% deltamethrin, Bayer Environmental Science, Research Triangle Park, NC, U.S.A.) [10]. Treatment is achieved by infusing/spraying ~4–6 g of the dust into individual prairie dog burrows across colonies [9]. Deltamethrin is a pyrethroid that directly affects fleas by opening sodium ion channels and causing hyperexcitation of flea nerve membranes, resulting in flea mortality [11].

Recently, fipronil, a phenylpyrazole, has been applied to prairie dog colonies in the form of bait (grain or pellets) to be eaten by prairie dogs [12–16]. Fipronil is a gamma-aminobutyric acid (GABA)-gated chloride channel antagonist, causing nerve/muscle hyperexcitation and mortality in fleas [17]. Prairie dogs excrete fipronil and metabolites (e.g., fipronil sulfone) in feces (i.e., fecal pellets) and, to a lesser extent, urine [18]. Prairie dogs routinely defecate and urinate in their burrows [19], transferring fipronil residues to that environment where flea larvae may be exposed and killed [18]. In most cases, deltamethrin or fipronil treatments effectively control fleas and suppress the transmission of *Y. pestis* with no known negative effects on prairie dogs or ferrets [5–7,20,21]. Deltamethrin is considered stable to photodegradation, with soil half-lives up to 209 d or more [22]. Fipronil degrades rapidly with exposure to ultraviolet (UV) light, but quite slowly in the absence of light (e.g., fipronil may persist for 200 d or more in darkness) [23]. Prairie dogs tend to nest >2 m underground and observations suggest UV light rarely penetrates more than ~31 cm into their burrows [24], indicating deltamethrin and fipronil (and metabolites) may persist for prolonged periods in these subterranean labyrinths [18].

Prairie dogs are keystone species and ecosystem engineers – more than 100 grassland species associate with them, including organisms that use prairie dog burrows as habitat, such as other rodents, insects, and amphibians [19,24–27]. Recent reports provide evidence for a negative effect of deltamethrin on survival of burrow-using deer mice (*Peromyscus* spp.) [3] and some arthropods found in prairie dog burrows (Eads et al. [Unpublished]). Despite this evidence and the potential for collateral effects of pulicides on non-target animals associated with prairie dogs, there is a paucity of work on species that co-occur on prairie dog colonies, particularly those that are commensal associates.

Adult amphibians (e.g., salamanders, toads) are commensal users of rodent burrows as refugia and hibernacula. Amphibians may seek refuge in burrows during their active periods (spring/summer) [28–30] or overwinter in burrows [31,32], where burrow environments reduce moisture loss and may facilitate prey acquisition. Amphibians have permeable skin and are typically in close contact with the substrate, thereby readily absorbing moisture and, perhaps, chemicals that may be present (i.e., dermal uptake) [33,34]. Pulicides are potentially toxic to adult amphibians [35]; for example, deltamethrin may contribute to oxidative stress [36,37] and is a potential neurotoxin when ingested by salamanders [38]. Fipronil is potentially permeable across adult amphibian skin, even at low application rates [34] and can disrupt metabolism [39].

In highly managed environments, such as most ferret reintroduction sites, collateral effects are an important consideration when developing conservation strategies and implementing disease mitigation actions [1,3]. Such consideration might be particularly appropriate in situations where commensal species (e.g., burrow-using amphibians) are also of conservation concern. The western tiger salamander (*Ambystoma mavortium*) is a species of mole salamander that uses burrows in prairie dog colonies [25] where fleas may be controlled using pulicides [1,5,6,8]. This salamander is one of several native amphibian species in the region that use burrows of different rodents as refugia and hibernacula (e.g., toads; *Anaxyrus punctatus*, *A. woodhousii*, and *A. boreas*) [40].

We assessed potential collateral effects of field-concentrations (i.e., realistic exposure amounts) of deltamethrin and fipronil residues on survival, body mass, and activity of western tiger salamanders in mesocosm “burrows”. We also assessed uptake of pulicide residues by quantifying the level of residue in gonad, liver, and whole-body tissues. We predicted 1) no acute effect of pulicide residues on salamander body mass or survival, 2) dermal uptake of pulicide residues as evidenced by measurable amounts of deltamethrin and fipronil (or metabolite) in salamander gonads, livers, and/or whole bodies, and 3) an effect of pulicides on salamander activity (i.e., time spent in burrows) (see Results Table 1).

**Table 1:** Predictions and outcomes for experiment assessing collateral effects of pulicides (deltamethrin and fipronil/fipronil sulfone) on tiger salamanders (*Ambystoma mavortium*). See text for statistical results.

Prediction	Outcome
Exposure to pulicides or pulicide residues does not cause acute effects on salamander body mass or survival.	All salamanders survived experiment. Treatments did not affect body mass.
Exposure to pulicides or pulicide residues results in dermal uptake as evidenced by measurable amounts of deltamethrin or fipronil (or its metabolite fipronil sulfone) in salamander tissues.	Deltamethrin and fipronil sulfone were detected in salamander tissues.
Exposure to pulicide or pulicide residues affects salamander activity (i.e., as an irritant and increasing activity or as a metabolic or locomotor depressor and decreasing activity). Time spent outside of burrows was defined as “active” and used as a proxy for “activity”.	Activity differed across treatments and was statistically significant, but might not represent biological importance.

## Materials and methods

### Study species

Western tiger salamanders (hereafter salamanders) are one of the largest terrestrial salamanders in North America (~15–22 cm in length as adults). They are relatively long-lived (> 10 years), reaching maturity at 4–5 years [41]. Although they breed in water, adults occupy a variety of terrestrial habitats including grasslands and open fields where they spend a significant portion of time in underground burrows constructed by rodents [4]. Rodent burrows provide these amphibians with refuge from hot and dry conditions in summer and provide hibernacula in winter with mild and relatively stable temperatures and humidity [42]. Adult salamanders are found commonly in burrows on prairie dog colonies, where the amphibians are rarely observed aboveground [25,43,44]. In addition to burrows providing shelter, prairie dog fecal pellets within and just outside the burrows attract arthropod prey for salamanders [24,25].

### Experimental design and implementation

Animal procedures were approved by the Institutional Animal Care and Use Committee of the USDA APHIS Wildlife Services National Wildlife Research Center (NWRC, Approval QA-3326), Fort Collins, CO, USA, with concurrence from the USGS Fort Collins Science Center Animal Use and Care Committee (Protocol 2021-04). We studied 29 salamanders, acquired commercially (*n *= 5; BackwaterReptiles.com) and from the Colorado Native Aquatic Species Research Facility (*n* = 24; Alamosa, Colorado) where salamanders are routinely live trapped to limit local amphibian incursion into clean facilities used for rearing boreal toads (*Anaxyrus boreas*). Salamanders were quarantined, treated, and monitored at the NWRC, Fort Collins, Colorado, USA, in the summer-fall of 2021.

**Fig 1:**
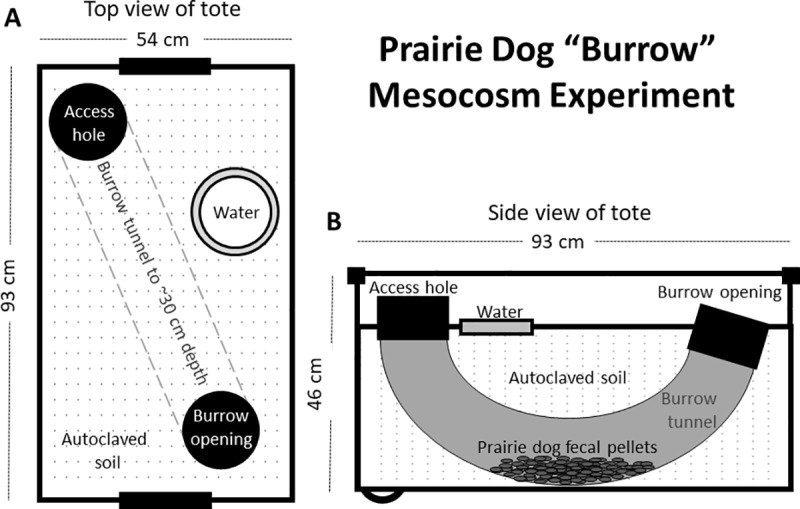
Schematic of mesocosm mimicking a prairie dog (*Cynomys* spp.) burrow. A. Top view; B. Side view.

Salamanders were housed individually in semi-natural mesocosms (151-L wheeled industrial plastic bins, Fig 1). Mesocosms were designed to mimic a natural rodent burrow typically used by salamanders. Each mesocosm held native Colorado soil (collected near NWRC and autoclaved), to a depth of ~ 50.8 cm, an artificial burrow made from flexible, corrugated plastic tubing (~61 cm long, 15.24 cm diameter, buried ~30.5 cm below the surface), and a dechlorinated water-filled bowl on the soil surface (Fig 1). Burrows had a surface opening (at the front) that angled down to the bottom and towards the back of the mesocosm where the tunnel protruded vertically to the soil surface and was covered with a cardboard cap. This setup provided investigator access to animals in the burrows for body mass and wellness checks. A moistened sponge was wedged into the plastic tubing at the top of the vertical end to humidify the burrow (under natural conditions, humidity at the depth of prairie dog nests has been measured at ~ 89–100%) [45]). Soil was pushed into each burrow such that the bottom was lined with soil along its full length.

**Fig 2:**
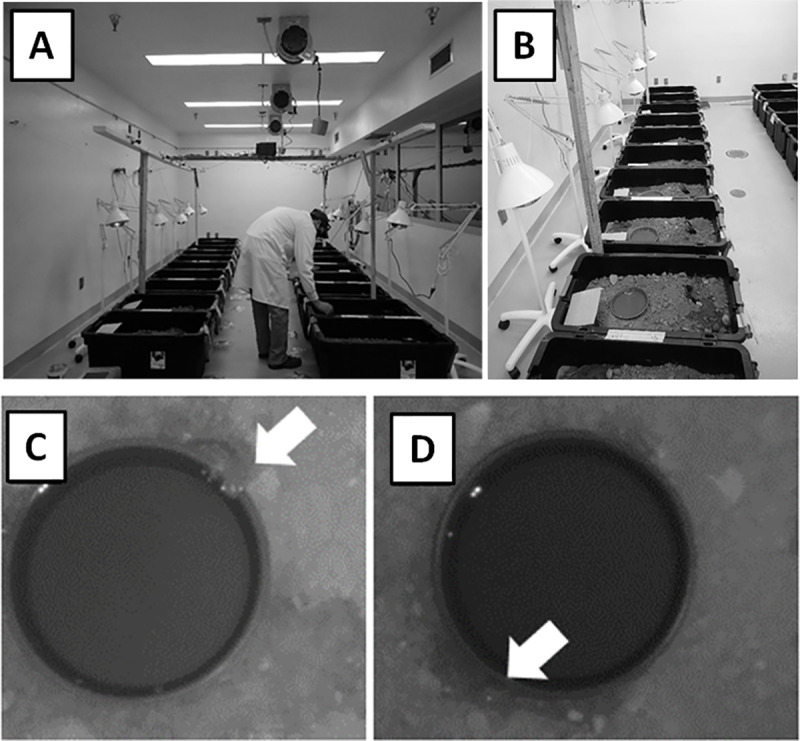
Laboratory setup. A. Temperature and light controlled experimental room; B. Mesocosms; C. Arrow indicates salamander visible on the surface of mesocosm; D. Arrow indicates salamander (head) partially visible under water bowl.

Mesocosms were assigned to 1) control: no pulicide and clean prairie dog fecal pellets (see below), 2) deltamethrin treatment: deltamethrin dust + clean prairie dog fecal pellets, or 3) fipronil treatment: fecal pellets procured from a prairie dog colony that was recently treated with fipronil-laced grain, where prairie dogs presumably had consumed the grain. The mesocosms were placed randomly in an array prior to treatments (Fig 2). Laboratory temperature was maintained at approximately 14–15 °C ambient (i.e., room) temperature, light was on a 14.5:9.5 h light:dark schedule (to mimic the approximate photoperiod experienced by the wild-caught salamanders), and heat lamps were on for 4 hours/day from 10:00–14:00, positioned over each neighboring pair of mesocosms in the array.

Upon accession at NWRC, body mass was determined for all salamanders (Adam CPWplus 6 scale with 2 g readability) and each salamander was evaluated for signs of parasites or disease. Salamanders were then placed randomly in individual, untreated mesocosms for a minimum of 7 days for quarantine. Following quarantine, salamanders were maintained in the untreated mesocosms for a period of 2 weeks (pre-treatment) beginning 15 July. On 29 July (start of treatment) salamanders were removed (< 1 minute) and mesocosms were treated (see below). The final experimental array included 8 control mesocosms, 11 mesocosms with deltamethrin dust, and 10 mesocosms with prairie dog fecal pellets laced with fipronil residues. The experimental exposure period ran for 63 d during which we monitored salamanders daily (using a portable endoscope as necessary to observe salamanders in burrows) and provided food three times per week (earthworms, crickets, wax worms, or meal worms). Water was replenished and cleaned as needed. We measured the mass of individual salamanders on the same day at approximately weekly intervals to assess potential changes in body mass.

#### Control.

Burrows in the control received 50 g of prairie dog fecal pellets collected aboveground, 1 year prior to the experiment. These fecal pellets were fresh (< 3 d old, brown and moist) when collected in the morning from prairie dog colonies in the Conata Basin, South Dakota that had never been treated with pulicides. These pellets were kept out of direct sources of UV light and were frozen (-18 °C) until use. We selected 50 g based on Wilcomb [24], who reported finding a “handful” of prairie dog fecal pellets in burrow nests when excavating prairie dog burrows in South Dakota. We tested five subsamples of these “clean” fecal pellets to calibrate the detection of fipronil residues in prairie dog fecal pellets from fipronil grain treated colonies ([45], see fipronil treatment below) (Liquid chromatography-mass spectrometry [LC-MS/MS], Analytical Toxicology Laboratory, Colorado State University, Fort Collins, CO).

#### Deltamethrin.

Burrows in the deltamethrin treatment received 1 g of DeltaDust^®^. The deltamethrin dust was applied by hand and sprinkled inside the opening and initial runway (~15 cm into the burrow) and out of direct light. The experimental dose was based on the amount of deltamethrin dust typically applied to individual prairie dog burrow openings in the field [5,9] which, based on the surface area of a typical burrow tunnel leading to a nest (14 m length, 7.5 cm radius) [24], is ~ 0.901 g of dust/m^2^. We used the dimensions of burrows in the mesocosms to determine the amount (1 g) of deltamethrin dust applied. In addition to deltamethrin dust, burrows in this treatment received 50 g of “clean” prairie dog fecal pellets from the same source as those used in the control.

#### Fipronil.

Burrows in the fipronil treatment received 50 g of prairie dog fecal pellets collected from a prairie dog colony in the Conata Basin, South Dakota where fipronil-laced grain bait (0.005% fipronil by weight; Scimetrics Limited Corp., Wellington, Colorado) was applied at ~ 50 g per prairie dog burrow opening ~1–3 d prior to collection of the fecal pellets [1]. These fecal pellets were collected and stored in an identical manner to the control pellets. We tested eight subsamples (i.e., 8 individual pellets) of these collected fecal pellets for fipronil residues using LC-MS/MS ([46]).

#### Tissue sampling.

On the final day of the experiment, salamanders were euthanized using MS222 [47]. Whole-body mass was determined, and gonads (ovaries and testes as available) and livers were removed from the carcasses using sterile dissection kits. All tissue was frozen (-80 °C) until analysis.

### Activity

We used custom-made open-source camera systems to monitor and quantify salamander aboveground activity relative to treatment. Cameras were mounted 1 m above neighboring pairs of mesocosms (Fig 2) and recorded high-resolution images (720p [1280 x 720 pixels]) in both visible (~300–700 nm) and near-infrared (NIR; > 700 nm; see results section) spectra of light every 15 min. Each system consisted of a camera mounted between two NIR light-emitting-diode (LED) illuminators that turned on and off via photoresistor when the laboratory room lights turned off and on (Model 4B, MakerFocus, www.makerfocus.com); a small single-board computer (Model Raspberry Pi 3b, Raspberry Pi Foundation Ltd., Cambridge, United Kingdom) that interfaced directly with the camera and recorded digital images; a removable, 32GB, solid-state memory device for storing images; and 3D-printed enclosures (designs available through Creative Commons open-source license at https://www.thingiverse.com/thing:922740, https://www.thingiverse.com/thing:3009532). Although some amphibians might be able to perceive NIR light in some circumstances [48], we designed our system to provide illumination at sufficient intensities and spectral bands to acquire images in the dark while minimizing the chance that salamanders could perceive the NIR light. The illuminators emitted light with a peak intensity at 850 nm, consumed 1 W of energy total, and were fitted with collimating lenses that diffused the light and formed a beam consistent with the 70° beam angle of the camera lens. Imagery recorded to the removable USB memory devices in.jpg format throughout the study was subsequently downloaded and analyzed through visual review using standard computer image software.

### Tissue analyses

Tissue samples from salamanders exposed to deltamethrin were analyzed at the U.S. Geological Survey Organic Chemistry Research Laboratory (Sacramento, California). Mass was determined for all samples upon arrival, then samples were stored at -20 °C. Prior to extraction, whole body (*n* = 11), liver (*n* = 11), and gonad (*n* = 9) samples (along with control/non-exposed samples of each; *n* = 2–3) were freeze dried and homogenized by Geno/Grinder^®^ (SPEX SamplePrep, Metuchen, New Jersey); in some cases where mass was greater, a portion of the sample was weighed out for extraction (target of 0.2 g dry weight; sample masses ranged from 0.0053 to 0.22 g, with 63.4 to 89.5% moisture). Samples were spiked with 50 µ L of 1 ng/ µ L of *cis*-permethrin-^13^C_6_ as a recovery surrogate, and then extracted using an EDGE^®^ (CEM Corporation, Matthews, North Carolina) [49] with 50:50 (v:v) hexane:acetone at 100 °C (hold time 3 min; total volume 30 mL). The extracts were exchanged into dichloromethane (0.5 mL), loaded onto carbon solid-phase extraction cartridges (Restek CarboPrep 90; 6 mL, 500 mg), then eluted with 10 mL of dichloromethane. The dichloromethane extracts were evaporated under nitrogen and exchanged into 0.2 mL acetonitrile. Prior to analysis 20 µ L of a 2.5 ng/ µ L bifenthrin-d_5_ was added as an internal standard and samples were filtered (0.45 µm PTFE). Quantitation for deltamethrin was done by gas chromatography-tandem mass spectrometry (GC-MS/MS) [49]. The limit of detection was 0.40 ng/g wet weight. Additional quality assurance/quality control QA/QC was achieved with one replicate sample (both samples had a non-detect), three procedural sand blanks (all non-detect), and one matrix spike of a whole salamander body (percent recovery of deltamethrin was 86.8%).

Tissue samples from salamanders exposed to fecal pellets from prairie dogs occupying a colony recently treated with fipronil-laced grain were tested for fipronil active ingredient and fipronil sulfone metabolite using LC-MS/MS testing at the Analytical Toxicology Laboratory, Colorado State University (Fort Collins, CO). Mass was determined for whole body (*n* = 10), liver (*n* = 10), gonad (*n* = 10), and control/non-exposed samples (*n* = 2 each) for calibration purposes prior to extraction. Whole body salamanders were freeze dried and homogenized with a Geno/Grinder. Whole body homogenate, liver, and gonad tissue samples (~100 mg) were homogenized and extracted with benchtop homogenizer (Beadbug, Benchmark Scientific, Sayreville, NJ). Tissues, 1 mL of 80% acetonitrile containing 10 ng/mL ^13^C_4_-fipronil and ^13^C_4_-fipronil sulfone, and 3 stainless steel beads were homogenized/extracted for 60 seconds at 4,000 rpm. Samples were centrifuged for 2 mins at 14,000 rpm; supernatant was added to a vial containing Agilent Universal dispersive solid phase extraction sorbent and then vortexed for 20 secs and centrifuged to pellet the sorbent. The final supernatant was transferred to an amber autosampler vial for LC-MS/MS analysis. Samples were analyzed for the presence of fipronil or fipronil sulfone with an Agilent 1290 UHPLC coupled to an Agilent 6460 triple quadruple mass spectrometer equipped with an Agilent Jet Stream electrospray ionization source (Agilent, Santa Clara, CA). Fipronil and fipronil sulfone were separated via chromatography on an Agilent Poroshell 120 C18 column (2.1 x 100 mm, 2.7 μm) held at 40° C. A sample volume of 5 μL was injected and a mixture of 70% water with 0.1% formic acid (A) and 30% acetonitrile with 0.1% formic acid (B) at a flow rate of 0.4 mL/min. The gradient elution used was 30% B to 80% B at 2 mins, then to 100% at 3.5 mins. The ionization source conditions were: nebulizer 40 psi; gas flow of 10 L/min at 300 °C; sheath gas flow of 11 L/min at 390 °C, and negative electrospray ionization. Two ion transitions (m/z) were monitored for each analyte and internal standard. The ion transitions monitored for fipronil were 435- > 330 and 250 m/z and 439- > 334 and 251 m/z for ^13^C_4_-fipronil. The ion transitions monitored for fipronil sulfone were 451- > 415 and 282 m/z and 455- > 419 and 286 m/z for ^13^C_4_-fipronil sulfone. Compound identifications were confirmed by retention time and the product ion ratios (± 20%). Data collection and processing were performed using Agilent MassHunter Quantitative software (v.B.08.01). Quantitation was performed with linear regression using 6-point calibration curves from 0.5 ng/g to 500 ng/g wet weight.

### Data analyses

#### Tissues and body mass.

We calculated summary statistics, by sample type, to illustrate general trends of pulicide residue uptake by salamanders.

We evaluated an effect of treatment on salamander body mass data using R version 4.1.2. We used generalized linear mixed models in R with a random effect for “salamander ID” to account for repeated measures during the before treatment and after treatment periods (‘lme4’ package and ‘glmer’) [50]. We considered fixed effects of treatment (control, deltamethrin, fipronil) and period (before or after treatment), and interactions between these variables.

#### Activity.

Cameras were active from 12 July through 30 September 2021. Images were examined on a computer by individual observers and each image was scored as the salamander being visible on the surface or not visible. A salamander was scored as observed (i.e., outside its burrow) if any portion of its body was visible in an image; salamanders in burrows were not recorded by the cameras.

We analyzed the camera data in R version 4.1.2 using a mixed modeling approach with a random effect for salamander ID (‘lme4’ package and ‘glmer’) [50]. We started with a binomial response for observed/non-detection of individual salamanders in each image. We analyzed the data under a before-after-control-impact (BACI) design, assessing potential temporal changes in aboveground observations from before to after treatment across each 24-hr period. The day the treatment was applied was excluded and considered a period of response to disturbance, and a period of potential acclimation to treatment. We considered fixed effects for treatment, period, hour, and all possible interactions, with a parsimonious model selected via backward elimination.

While examining images, observers noted that 13 salamanders were sometimes seen under the water bowl, with portion(s) of their bodies (e.g., tail) visible in images(s). For the above analyses, these events were scored as observed “aboveground”. Because observations of salamanders on the surface but under the water bowl could be interpreted differently from observations on the surface and away from the bowl, we assessed the data with this additional category of “under the bowl”, for the 13 salamanders with evidence of this behavior. We ran mixed models with a binomial response for observed/non-detection of individual salamanders under their water bowl and fixed effects for treatment, period, hour, and all possible interactions. We selected a parsimonious model via backward elimination. Cardboard caps occasionally came off such that salamanders were sometimes observed on the sponge; caps were replaced within 24 hours. We did not record those observations as detections because the salamanders were technically still in the burrows but on the sponge.

## Results

None of the salamanders exhibited signs of disease or distress upon arrival in the laboratory and all survived the experiment. We found no acute effects from the pulicide treatments and treatments did not impact body mass, as predicted. Also as predicted, we detected both pulicides in salamander tissues. Overall, salamanders spent the vast majority (98%) of the time inside the burrows, but there were some potential differences in activity levels, supporting our prediction of an effect of pulicides on activity (Table 1).

### Experimental design and implementation

Subsamples (i.e., individual pellets) of clean prairie dog fecal pellets (collected at colonies not treated with pulicides) yielded no fipronil or fipronil sulfone when assayed. All subsamples (i.e., individual pellets) of fecal pellets from prairie dogs exposed to fipronil-laced grain bait that were assayed yielded fipronil (range: 459.80–2766.30 ng/g) and fipronil sulfone (range: 393.65–848.00 ng/g). The mean of all eight pellets was 1,019.89 [SE = 302.93] ng/g fipronil and 611.96 [SE = 48.612] ng/g fipronil sulfone, confirming that there was potential exposure of salamanders to these residues in the fipronil treatment. We did not observe evidence of salamanders biting (or consuming) prairie dog fecal pellets, suggesting pulicide residue exposure would occur via external routes (e.g., dermal absorption).

### Body mass

Most of the salamanders gained body mass over the course of the experiment, a few lost, and two remained the same. The mixed model did not support evidence for an effect of treatment on body mass.

### Tissue

We assayed gonads, liver and whole bodies for deltamethrin and fipronil (and metabolites). Two gonads and one liver (3 separate individuals) were destroyed on dissection and not assayed. Neither deltamethrin nor fipronil (nor metabolites) were detected in gonad, liver, or whole-body samples in salamanders from the control. Detections of pulicide residues were most frequent for salamanders in the deltamethrin treatment. Deltamethrin was detected in 4/9 gonads (0.4 to 2.7 ng/g), 1/11 livers (1.6 ng/g), and 9/11 whole bodies (0.4 to 3.8 ng/g) in the salamanders exposed to deltamethrin. Fipronil was not detected in the tissues of salamanders in the fipronil treatment, but fipronil sulfone was detected in 3/10 gonads (1.2 to 7.4 ng/g), 1/9 livers (11.9 ng/g), and 1/10 whole bodies (7.8 ng/g).

### Activity

Images were successfully recorded for salamanders in 7 control, 10 deltamethrin, and 8 fipronil treatments. One salamander (deltamethrin treatment) was never observed via camera. However, this individual was monitored and confirmed to be alive throughout the experiment; therefore, we included data from this individual in the analyses. Ten cameras failed to record an image on 1–16 individual occasions (overall 73 images were missed across 10 individual salamanders) but the total number of images per salamander was very similar by treatment and period. Four cameras malfunctioned such that no images were recorded for two salamanders in the fipronil treatment and only post-treatment images (i.e., no pre-treatment images) were recorded for one salamander in the control and one salamander in the deltamethrin treatment. The camera for one of the control salamanders started recording a day late, but those data were included. For these 25 salamanders, we assessed 181,579 images (1,536-1,588 pre-treatment images/salamander and 5,975-5,983 post-treatment images/salamander). In images, salamanders were either on the surface and visible or, in some cases, on the surface and partly visible (i.e., a portion of its body was under the water bowl), or not visible and presumably in the burrow. We considered detections of salamanders on the surface of the mesocosm as a proxy for time spent outside of the burrow (i.e., time away from pulicide residues).

Salamanders were visible on the surface of the mesocosm in only 2% of all images (*n* = 3,574), but 24 of 25 salamanders were visible on the surface in at least one image. In the mixed model analysis, the following interactions were unsupported and eliminated: treatment × period × hour (χ^2^ = 0.404, *df* = 2, *P* = 0.817), period × hour (χ^2^ = 0.334, *df* = 1, *P* = 0.564), and treatment × hour (χ^2^ = 1.808, *df* = 2, *P* = 0.405). The treatment × period interaction (χ^2^ = 96.117, *df* = 2, *P* < 0.001) and hour (χ^2^ = 105.797, *df* = 1, *P* < 0.001) were supported (S1 Table). Aboveground observations increased, in both treatments and the control, albeit weakly, from the pre-treatment period to after treatment. The increase was stronger for salamanders in the deltamethrin treatment (1–3% of images) than for individuals in the control or fipronil treatment (1–2% and 0–1%, respectively) (Table 2).

**Table 2:** Percent of above-ground observations (i.e., number of images where salamanders were visible on the surface of the mesocosm) for experiment assessing the collateral effects of pulicides (deltamethrin and fipronil/fipronil sulfone) on tiger salamanders (*Ambystoma mavortium*).

Treatment	Pre-Treatment	Post-Treatment	Difference
Control	0.68	1.95	1.27
Deltamethrin dust	0.95	2.98	2.03
Fipronil/ fipronil sulfone fecal pellets	0.03	1.94	1.95

The analysis of observations under the water bowls before and after treatments included 13 salamanders (6 control, 2 deltamethrin, 5 fipronil) and 98,308 images (1,536-1,588 images/salamander pre-treatment and 5,975-5,983 images/salamander post-treatment). The control salamander with a 1 day late start date was included in this analysis. The number of observations of salamanders under the water bowls was low (*n* = 2,723, ~ 3% of images) but represented 1–97% (*x̄* = 53%) of aboveground observations. The treatment × period × hour interaction was removed (χ^2^ = 3.280, *df* = 2, *P* = 0.194). The following interactions were supported (S2 Table): treatment × period (χ^2^ = 181.60, *df* = 2, *P* < 0.001), treatment × hour (χ^2^ = 8.094, *df* = 2, *P* = 0.017), and period × hour (χ^2^ = 14.398, *df* = 1, *P* < 0.001). The number of observations of salamanders under the water bowls remained similar (i.e., was not statistically significant) from pre- to post-treatment for the deltamethrin treatment (1%) but increased from pre- to post-treatment for the control and fipronil treatment (both from 0 to 2%). This behavior was infrequent and remained similar by hour in the pre-treatment period but increased in frequency (98% of images) and was most often observed around 0000 h in the post-treatment period.

Deltamethrin concentrations in whole body samples correlated positively with the amount of time those salamanders were presumably in the burrows (i.e., not observed) during the post-treatment period (*r*^2^ = 0.60, *F* = 7.563, *df* = 1, *P* = 0.040). Fipronil and fipronil sulfone were detected in too few samples for a similar analysis.

## Discussion

Pulicides were delivered to mesocosm habitats in concentrations, and with application methods, that were intended to mimic the use of these pulicides in the field to control fleas and mitigate plague on colonies of prairie dogs. In the field, deltamethrin dust is introduced directly to burrows and fipronil residues are introduced indirectly to burrows via fecal pellets from prairie dogs that have eaten fipronil-laced bait above ground [9,18]. After treatments, salamander tissue (gonad, liver and whole body) showed evidence of deltamethrin and fipronil sulfone as suggested by Van Meter et al. [34]. This indicates that treatment methods were effective at exposing salamanders to pulicides and that uptake of pulicides can occur in salamanders inhabiting prairie dog burrows at pulicide-treated colonies. Uptake of pulicide residues presumably occurred via dermal absorption, as demonstrated previously with deltamethrin in Asiatic toads (*Bufo bufo gargarizans*) [51] and with fipronil in several toad and frog species [34]. Based on the number of animals, fipronil uptake appears limited relative to deltamethrin under our experimental conditions. We remind readers that the exposure concentration per salamander is unknown for either pulicide. While fipronil sulfone concentrations were higher than observed deltamethrin concentrations in salamander tissue, the relative impact of the two pulicides is unknown.

While we aimed to provide the best approximation of a field scenario as possible, it was not exact. For example, our application method for deltamethrin (i.e., sprinkling dust directly inside mesocosm burrow openings) may represent a higher rate of exposure relative to burrow infusions under field conditions because manual application may result in clumping of the dust relative to infusion. Fipronil exposure was potentially on the low side of concentrations possible in prairie dog fecal pellets because our treatment pellets were collected relatively early after post-treatment such that prairie dog feces may not have achieved maximum fipronil (and metabolite) concentrations. Other rodents occurring in prairie dog burrows could also contribute to the presence of fipronil sulfone if they had ingested the fipronil-laced grain. Additionally, in the field, prairie dogs or other commensal organisms (e.g., mice) may transfer and move deltamethrin dust within burrows, which might affect the distribution of the dust in the burrow and subsequently affect salamander exposure. Prairie dogs were not included in our treatments.

In general, salamander activity patterns and surface detections were consistent with activity patterns observed at prairie dog colonies in the field (DAE, personal observation), with rare above ground observations. Similarly, in our experiment, salamanders spent the vast majority of time in burrows; however, they spent statistically less time in burrows following both pulicide treatments. The change was most pronounced immediately after treatments, and the effect was more notable in the deltamethrin treatment, although salamanders with higher concentrations of deltamethrin in whole body tissues spent more time in burrows (i.e., were observed less often aboveground). The result of less time spent in a burrow could be interpreted as salamanders avoiding deltamethrin, perhaps to reduce irritation or other negative consequences of contact with the pulicide. Additionally, spending less time in treated burrows might indicate behavioral change potentially affecting salamander physiology (e.g., increased heat stress or water imbalance) or increasing predation risk. Alternatively, disturbance related to the experiment, daily checks and periodic weighing, could also have precipitated movement. Overall, while we did find a statistically significant treatment effect on activity, it is unknown whether the impact was biologically meaningful.

We found salamanders in the deltamethrin treatment using an alternate retreat site (under water bowls) during daylight hours more frequently than individuals in the control or fipronil treatments. However, if the salamander was completely under the water bowl, they were undetectable, and that image was scored as “in the burrow”, thus we do not have an accurate count of how often salamanders used the water bowl as retreat site (for controls or treatments).

A number of factors could affect the exposure of salamanders to either of the pulicides. For example, movements of resident prairie dogs or any commensal organisms into or out of the burrow could affect the amount or availability of pulicide in the burrow. Additional work considering differences in absorption rates in burrow environments for different species, and the specific effects of pulicides on ion transport across amphibian skin could be useful. Future studies of fipronil could consider the effects of time between fipronil application and fecal pellet collection on the amount of fipronil residue in prairie dog fecal pellets that is available to organisms in burrow environments.

The presence of pulicide residues in multiple tissues suggests the potential for long-term effects on salamander health and survival [52,53], including effects on metabolism and hormones [32,52], disease dynamics [54–56], and reproduction [57,58]. Detections of deltamethrin and fipronil in whole bodies or organs were not associated with salamander death or obvious signs of distress (although changes in activity could be considered a sign of stress), potential long-term effects from pulicide residue exposure in salamanders are of concern for several reasons. First, flea control measures are often completed annually at core conservation sites for prairie dogs and black-footed ferrets [5,6,8]; second, salamanders may survive >10 years under natural conditions [41], thus accumulation of pulicides in tissue is a possibility; and third, mole salamanders (i.e., tiger salamanders) do not typically move great distances (e.g., maximum of ~ 285 m [59–61]).

Field studies are needed to determine if our results apply under natural conditions. In particular, interactions between salamanders and prey items are important components to field trials because deltamethrin dust and fipronil bait treatments might reduce the abundance of arthropods within and/or outside prairie dog burrows, including salamander prey items (e.g., grasshoppers [42]). Salamanders implement a “sit-and-wait” ambush predatory strategy, most commonly feeding on live prey, suggesting they might not consume dead arthropods (or other organisms) killed by pulicide residues. While this could reduce oral exposure (and potential toxic effects [38]), both deltamethrin and fipronil residues cause hyperexcitation of insect nerve membranes, such that dying, and sometimes long-dead, insects still move (DAE, personal observations), and such movements could attract salamanders and potentially increase oral exposure. Reductions in arthropod prey availability due to pulicide residues might stress resident salamanders by limiting food/water resources (similar ideas have been proposed for small mammals at flea control sites [3]).

Our results illustrate a lack of acute effects of deltamethrin dust or fipronil residues on salamander survival but indicate the potential for long term effects. This work is broadly applicable as there is evidence of widespread commensal relationships between ambystomatid salamanders and the many plague-susceptible rodents [4,28] that are sometimes treated with pulicides for flea control and plague mitigation [1]. Experiments in these systems focusing on salamanders or other amphibians (e.g., long-lived toads) and other non-target animals (e.g., arthropods, reptiles, birds) would be useful to further assess the potential for collateral effects from pulicide residues that are used for operational disease mitigation to promote wildlife conservation. Our research provides wildlife managers information that can be used to weigh conservation advantages of pulicide treatments to target species versus risks of pulicide treatments to non-target organisms.

## Supporting information

S1 Table Generalized linear model of salamander aboveground activity. Coefficient estimates and standard errors (*SE*s) for a generalized linear mixed model evaluating effects of hour of day (24 hr clock), treatment (deltamethrin or fipronil residues, with control [no residues] as a baseline), and period of experiment (before treatment, with after treatment as a baseline) on tiger salamander aboveground activity, outside burrow mesocosms. The model included a random effect for “salamander ID” to account for repeated measures from individual salamanders over time. The values are from a model developed using backward elimination such that only statistically significant variables are included.
(DOCX)

S2 Table Generalized linear model of salamander use of water bowls as cover. Coefficient estimates and standard errors (*SE*s) for a generalized linear mixed model evaluating effects of hour (24 hr clock), treatment (deltamethrin or fipronil residues, with control [no residues] as a baseline), and period of experiment (before treatment, with after treatment as a baseline) on tiger salamander use of water bowls as cover when outside burrow mesocosms. The model included a random effect for “salamander ID” to account for repeated measures from individual salamanders over time. The values are from a model developed using backward elimination such that only statistically significant variables are included.
(DOCX)
