# Supplementary material for: Assessing potential collateral effects on amphibians from insecticide applications for flea control and plague mitigation
Source: PLoS One. 2025 May 12;20(5):e0320382. doi: 10.1371/journal.pone.0320382 (PMC12068567; doi:10.1371/journal.pone.0320382)
Supplement: S1 Table — Coefficient estimates and standard errors (SEs) for a generalized linear mixed model evaluating effects of hour of day (24 hr clock), treatment (deltamethrin or fipronil residues, with control [no residues] as a baseline), and period of experiment (before treatment, with after treatment as a baseline) on tiger salamander aboveground activity, outside burrow mesocosms. The model included a random effect for “salamander ID” to account for repeated measures from individual salamanders over time. The values are from a model developed using backward elimination such that only statistically significant variables are included. (DOCX) [file pone.0320382.s001.docx]

Supplementary Materials:

Assessing potential collateral effects on amphibians from insecticide applications for flea control and plague mitigation

David A. Eads^1*^, Susan A. Shriner^2^, Jeremy W. Ellis^2^, Paul M. Cryan^1^, Michelle L. Hladik^3^, Gregory P. Dooley^4^, Erin Muths^1^

^1^ U.S. Geological Survey, Fort Collins Science Center, Fort Collins, Colorado, United States of America

^2^National Wildlife Research Center, U.S. Department of Agriculture, Animal and Plant Health Inspection Service, Wildlife Services, Colorado, United States of America

^3^U.S. Geological Survey, California Water Science Center, Sacramento, California, United States of America

^4^Department of Environmental and Radiological Health Sciences, College of Veterinary Medicine and Biomedical Sciences, Colorado State University, Fort Collins, Colorado, United States of America

*Corresponding author

E-mail: deads@usgs.gov

**Table S1.** **Generalized linear model of salamander aboveground activity.** Coefficient estimates and standard errors (*SE*s) for a generalized linear mixed model evaluating effects of hour of day (24 hr clock), treatment (deltamethrin or fipronil residues, with control [no residues] as a baseline), and period of experiment (before treatment, with after treatment as a baseline) on tiger salamander aboveground activity, outside burrow mesocosms. The model included a random effect for “salamander ID” to account for repeated measures from individual salamanders over time. The values are from a model developed using backward elimination such that only statistically significant variables are included.

| **Effect** | **Coefficient** | ***SE*** |
| --- | --- | --- |
| Intercept | -3.639 | 0.042 |
| Hour | -0.0003 | 0.00002 |
| Treatment (deltamethrin) | 0.435 | 0.042 |
| Treatment (fipronil) | -0.002 | 0.048 |
| Period (before) | -1.061 | 0.113 |
| Treatment (deltamethrin) × period (before) | -0.095 | 0.145 |
| Treatment (fipronil) × period (before) | -3.227 | 0.589 |
